# Organic-Inorganic Hybrid Nanoparticles for Bacterial Inhibition: Synthesis and Characterization of Doped and Undoped ONPs with Ag/Au NPs

**DOI:** 10.3390/molecules20046002

**Published:** 2015-04-07

**Authors:** Carlos Alberto Huerta Aguilar, Adriana Berenice Pérez Jiménez, Antonio Romero Silva, Navneet Kaur, Pandiyan Thangarasu, Jorge Manuel Vázquez Ramos, Narinder Singh

**Affiliations:** 1 Facultad de Química, Universidad Nacional Autónoma de México (UNAM), Ciudad Universitaria, Coyoacán, 04510 México D.F., Mexico; E-Mails: waitthinkandfast@hotmail.com (C.A.H.A.); remin_1711@hotmail.com (A.B.P.J.); antromsil@comunidad.unam.mx (A.R.S.); 2 Centre for Nanoscience & Nanotechnology, Panjab University, Chandigarh, Panjab 160014, India; E-Mail: navneetkau@pu.ac.in; 3 Department of Chemistry, Indian Institute of Technology Ropar, Rupnagar, Panjab 140001, India; E-Mail: narindarchem@gmail.com

**Keywords:** antibacterial studies, organic nanoparticles, lipoic acid, *Staphylococcus aureus*, *Bacillus cereus*, *Escherichia coli*, *Salmonella typhi*

## Abstract

Organic nanoparticles (ONPs) of lipoic acid and its doped derivatives ONPs/Ag and ONPs/Au were prepared and characterized by UV-Visible, EDS, and TEM analysis. The antibacterial properties of the ONPs ONPs/Ag and ONPs/Au were tested against bacterial strains (*Staphylococcus aureus*, *Bacillus cereus*, *Escherichia coli* and *Salmonella typhi*). Minimal Inhibitory Concentration (MIC) and bacterial growth inhibition tests show that ONPs/Ag are more effective in limiting bacterial growth than other NPs, particularly, for Gram positive than for Gram-negative ones. The order of bacterial cell growth inhibition was ONPs/Ag > ONPs > ONPs/Au. The morphology of the cell membrane for the treated bacteria was analyzed by SEM. The nature of bond formation of LA with Ag or Au was analyzed by molecular orbital and density of state (DOS) using DFT.

## 1. Introduction

The use of diverse nanomaterials as antibacterial agents is growing due to their small size, which ensures desirable contact with bacterial cells; in particular, harmless materials that can effectively inhibit bacterial growth is potentially important for preserving packaged food, pharmaceutical and cosmetic products [1]. However, most of the studies are focused on inorganic nanoparticles such as AgNPs [2,3,4,5,6], AuNPs [7,8,9], CuNPs [10,11], ZnONPs [12,13,14], TiO_2_ [15], ZnO/Ag, TiO_2_/Cu or Ag/Au NPs [16,17,18,19,20,21] as antibacterial agents. Yet some of these materials are toxic [22,23], expensive [24] or hard to apply due to their low stability [25,26].

Although inorganic-based NPs are effective at inhibiting bacterial growth, their accumulation in biological tissues or in food materials can cause serious problems (damage vital organs) as they are non-biodegradable. On the other hand, organic molecules such as ampicillin, amoxicillin, benzylpenicilllin, bleomycin, cefamandole, levofloxacin, clarithromycin, lincomycin and tetracyclines (antibiotics), and amphotericin, griseofulvin, metronidazole (antifungals) have been shown as efficient antibacterial or anti-viral agents. The accumulation of these molecules in biological systems is much lower and they are easily biodegradable. Thus, the study of nanoparticles based on organic compounds (ONPs) has become an interesting topic. Nevertheless, reports on ONPs in the literature are limited, and most of them are focused on polymers, such as chitosan NPs [27,28,29]. The electronic/optical properties of ONPs are important because their van der Waals intermolecular forces, hydrogen-bonding interactions, and electrostatic attractions are fundamentally different from those of inorganic metals or semiconductors [30]. The changes in their physical and chemical properties increase exponentially with the surface area from NPs to bulk materials [31,32]

α-Lipoic acid (LA), which is present in eukaryotic and prokaryotic cells [33], functions as a cofactor for mitochondrial α-ketoacid dehydrogenases, and it forms a “universal antioxidant” redox couple with dehydrolipoic acid (DLA) [34]. LA plays a key role in the regeneration of biological antioxidants such as vitamin C (D/L-ascorbic acid) and vitamin E [35], sequestration of Reactive Oxygen Species (ROS), regulation of redox processes, and manipulation of gene expression and apoptosis [35]. Several authors have studied the catalytic and biological applications of LA because of its biocompatibility [36,37,38,39,40], and its use as a biocompatible-stabilizing agent [41,42]. The presence of a disulfide (dithiolane ring) in the LA structure facilitates an interaction with metallic NPs, especially with AuNPs and AgNPs [43], which can enhance their antibacterial effects. In the literature, the antibacterial properties of ONPs (lipoic acid) and their nanohybrids ONPs/Ag and ONPs/Au have not been reported yet. Therefore, the present study aimed to evaluate the antimicrobial activity of ONPs, ONPs/Ag and ONPs/Au against four different Gram-positive and Gram-negative bacteria. Furthermore, the toxicity of the organic molecule or its binding with metal is closely related to the structural and electronic parameters, thus the geometrical and electronic properties of LA are analyzed by Density Functional Theory (DFT) in order to support the antibacterial effect.

## 2. Results and Discussion

**Figure 1:**
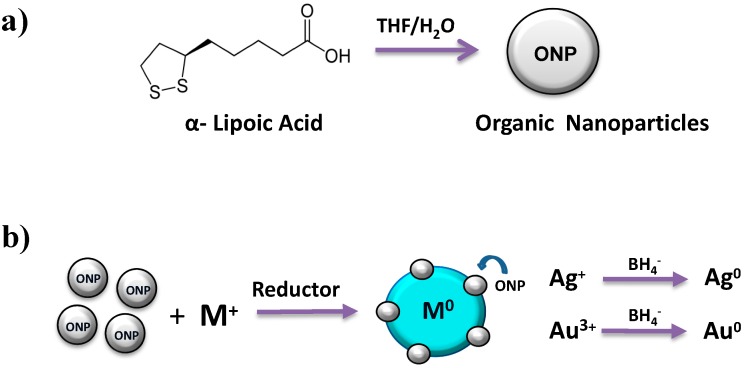
(**a**) Formation of α-lipoic acid ONPs; (**b**) preparation of ONPs/Ag and ONPs/Au.

Aqueous colloidal suspensions of ONPs (α-lipoic acid) and the hybrids ONPs/Ag and ONPs/Au were prepared (see Figure 1a,b). All the NPs were characterized by UV-visible spectrophotometry, Transmission Electron Microscopy (TEM) [44] and Energy Dispersive X-Ray Spectroscopy (EDS) [44].

**Figure 2:**
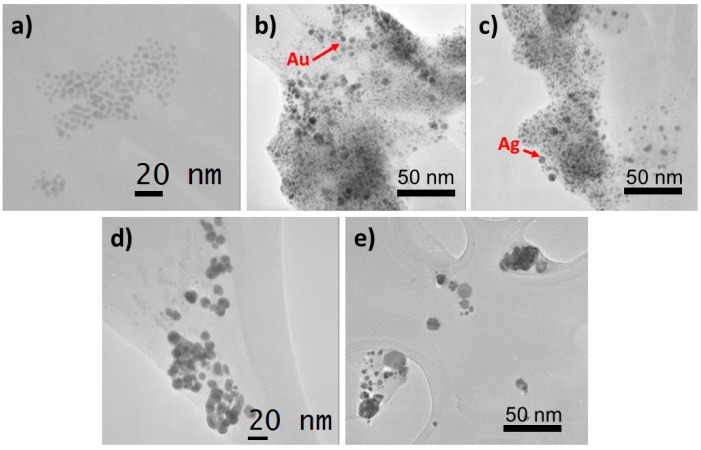
Transmission Electron Microscope micrographs: (**a**) ONPs; (**b**) ONPs/Ag (ratio 10:1); (**c**) ONPs/Au (ratio 10:1); (**d**) Ag NPs and (**e**) Au NPs.

The size and morphology of the samples were determined by TEM (25,000× magnification) to provide a representative number of particles [45] (Figure S1). The data (Figure 2) show that ONPs are round shaped (~5.0 nm) (Figure 2a), and ONPs/Ag and ONPs/Au appear as dark particles due to the existence of high density Ag or Au atoms (Figure 2b,c), in agreement with a published report [46]. The particle diameters and size distributions of ONPs/Ag (~13.0 nm) and ONPs/Au (~8.0 nm) are smaller than those of the analogous AgNPs (Figure 2d) and AuNPs (Figure 2e) and the particles are of different sizes, however, a uniform size was obtained after preparing hybrid ONPs/Ag or ONPs/Au (Supplementary Material S1). 

This suggests that ONPs act as a size controlling agent and prevent the aggregation of metallic nuclei. It was observed that the ONPs/Au particles are more homogeneous than ONPs/Ag.

**Figure 3:**
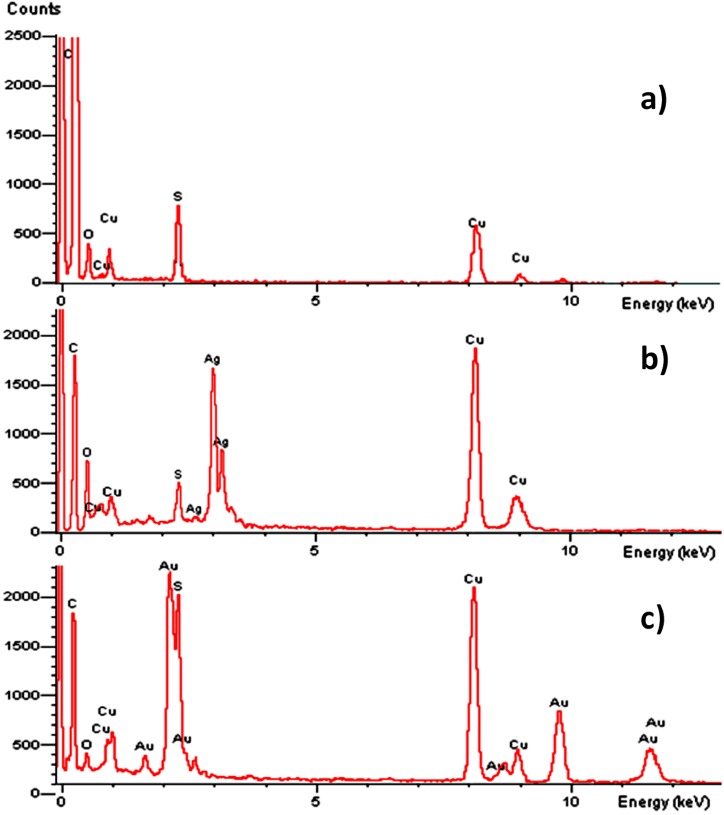
Energy Dispersive Spectrometry analysis: (**a**) ONPs, (**b**) ONPs/Ag (ratio 10:1), (**c**) ONPs/Au (ratio 10:1).

The EDS study was performed by sampling at 15 different spots in the TEM analyses grid, and the results (Figure 3) show the presence of C, S and O in the ONPs. A signal that corresponds to C, confirms the organic nature of ONPs, however, the quantification of the carbon content is difficult due to interference from the carbon coated grid in the TEM and EDS analysis. The presence of Ag or Au in ONPs/Ag or ONPs/Au is also confirmed by the TEM-EDS. The signal related to Cu commonly appears in EDS because of its presence in the grid.

### 2.1. Optical Properties of ONPs, ONPs/Ag and ONPs/Au

**Figure 4:**
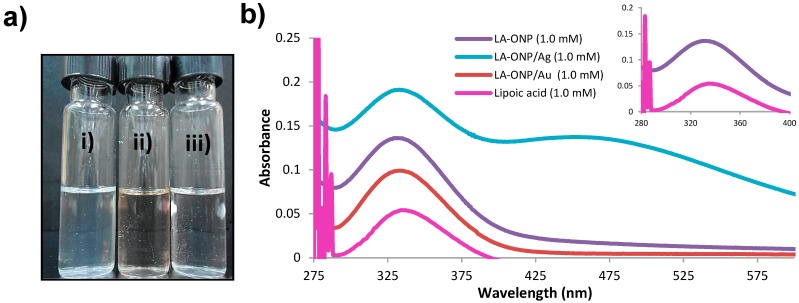
Characterization of ONPs; (**a**) visible appearance: (**i**) ONPs; (**ii**) ONPs/Ag and (**iii**) ONPs/Au; (**b**) UV-Visible profile of lipoic acid-ONPs.

The band at 333 nm in the UV-visible absorption spectrum for lipoic acid in THF (Figure 4), also appeared with high intensity in the ONPs [31,32]. The spectral behavior of LA-ONP/Ag is significantly modified compared to that of ONPs (Figure 4) showing a high intensity peak (333 nm) along with a shoulder band (~475 nm). This indicates the existence of a strong interaction between metallic NPs and ONPs.

### 2.2. Antibacterial Activities of ONPs, ONPs/Ag and ONPs/Au

**Figure 5:**
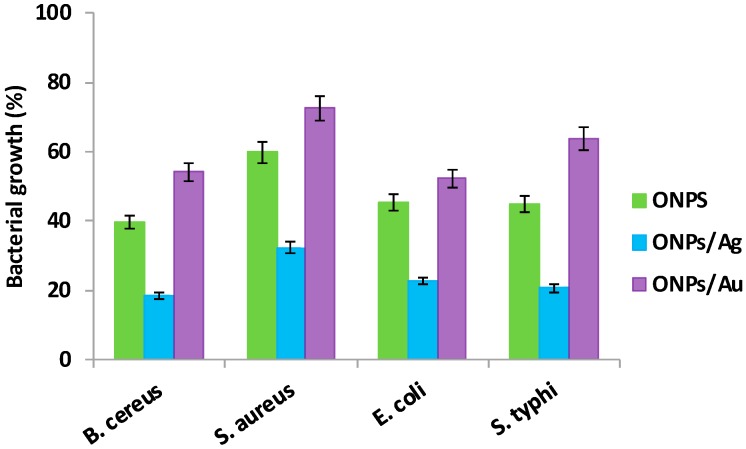
Antibacterial effects: Bacterial survival (%) after treatment with different NPs (0.2 mM).

The antibacterial properties of ONPs, ONPs/Ag and ONPs/Au were evaluated against Gram positive (*Staphylococcus aureus* and *Bacillus cereus*), and Gram negative (*Escherichia coli* and *Salmonella typhi*) bacteria. Results (Figure 5, Table 1) show that ONPs, ONPs/Ag and ONPs/Au have considerable inhibitory effects against both Gram positive and Gram negative bacteria.

**Figure 6:**
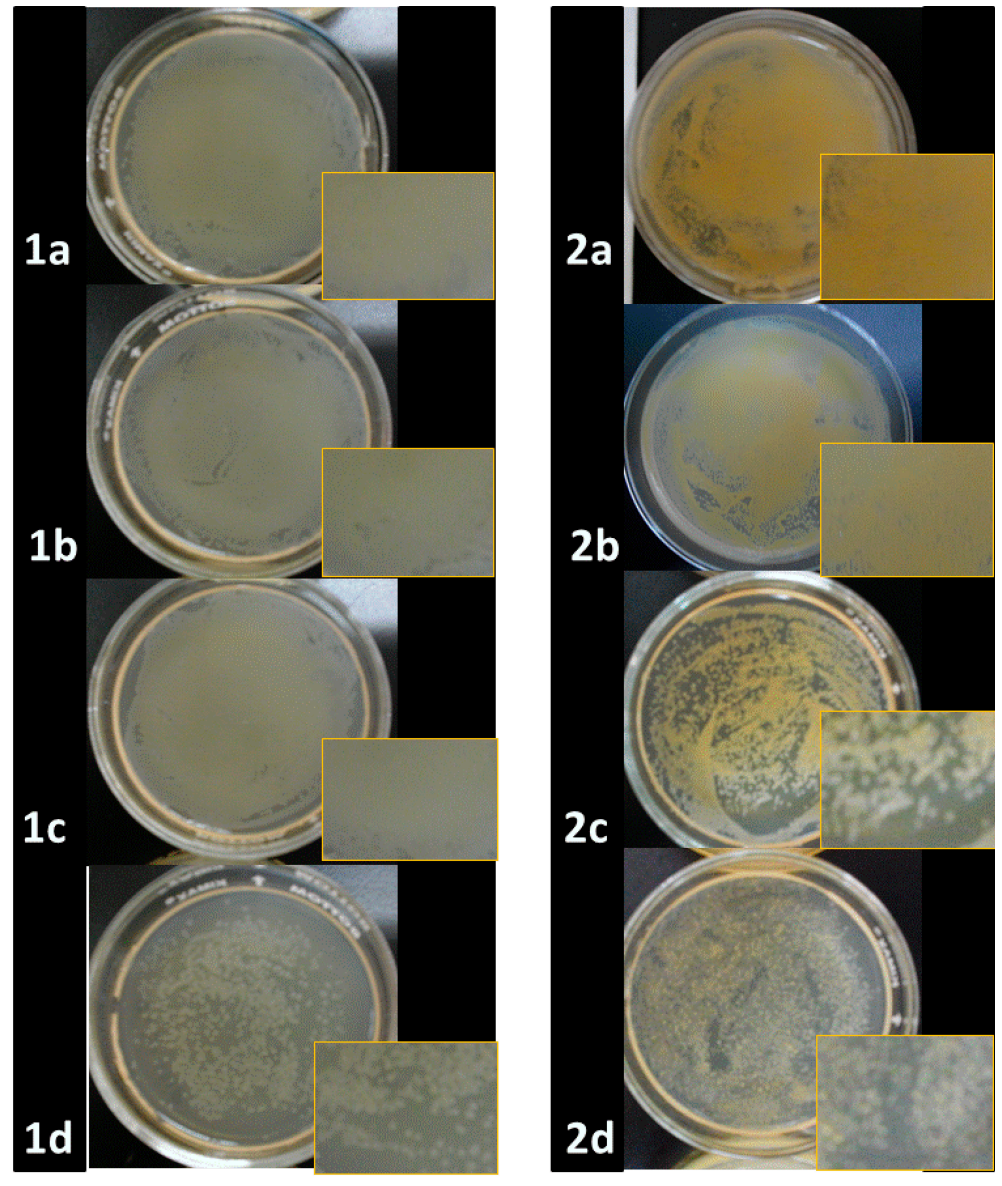
Comparative bacterial growth inhibition (**1**) *Salmonella typhi* and (**2**) *Staphylococcus aureus:* after treatment (**a**) Control; (**b**) AgNPs (0.02 mM); (**c**) ONPs (0.2 mM); (**d**) ONPs/Ag (0.2 mM).

The qualitative tests (Figure 5) reveal the following inhibition trend: ONPs/Ag > ONPs > ONPs/Au. Furthermore, the number of viable microorganisms decreases when NPs are added to the medium (Figure 6 and Figure S2). Figure 6 describes only the growth rate of bacteria for the different types of NPs. After addition of NPs onto the surface of the Mueller Hinton agar-filled plates, these were then shaken completely in order to get a uniform distribution of NPs in the solution for the bacterial culture at 36 °C for 36 h. The greatest difference between the control (1a, 2a) and treated samples was seen for ONPs/Ag (1d, 2d), so the inhibition effect is higher for ONPs/Ag than for ONPs (1c, 2c) or Ag NPs (1b, 2b), indicating that there is a synergic effect between ONPs and AgNPs.

**Table 1:** Minimal Inhibitory concentration of different bacteria treated NPs.

Inhibitor	Minimal Inhibitory Concentration (MIC) [44] [mmol/L]			
	Gram (+)		Gram (−)	
	*Bacillus. cereus*	*Staphylococcus aureus*	*Escherichia coli*	*Salmonella typhi*
**ONPs**	0.31 ± 0.04	0.39 ± 0.03	0.39 ± 0.05	0.39 ± 0.04
**ONPs/Ag**	0.26 ± 0.05	0.26 ± 0.05	0.22 ± 0.02	0.22 ± 0.03
**ONPs/Au**	0.39 ± 0.11	0.44 ± 0.02	0.44 ± 0.09	0.44 ± 0..04
**Ag-NPs**	0.17 ± 0.04	0.17 ± 0.03	0.13 ± 0.01	0.13 ± 0.05
**Au-NPs**	1.76 ± 0.05	1.82 ± 0.05	1.28 ± 0.07	1.12 ± 0.09

After measuring bacterial growth at different concentrations (Figure S3), it was observed that the antibacterial effectiveness greatly increases with increasing concentration of NPs. For all ONPs (undoped or doped), there is no significant difference in optical density at low concentrations (0.05 to 0.2 mM); but cell death occurs at higher concentration, indicating a possible perturbation of the cytoplasmic membrane (see SEM analysis) [47].

**Figure 7:**
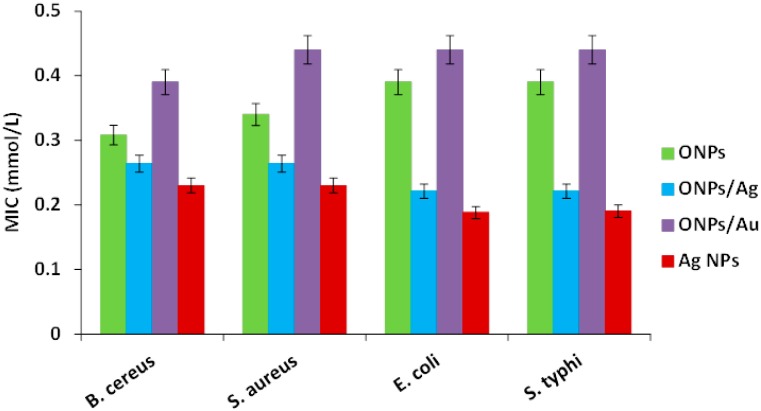
Minimal inhibitory concentration (MIC) of bacteria treated with different NPs.

The MIC values show that ONPs are more effective in growth control for Gram-positive than for Gram-negative bacteria (Figure 7), and they indicates the minimum amount of NPs required to avoid bacterial growth (mmol/L). This behavior can be explained by the fact that Gram-negative species possess an outer membrane, which acts as an intrinsic barrier for hydrophobic agents due to the presence of negatively charged phosphate in lipopolysaccharides (LPS) [48] along with hydrophobic lipid elements. In contrast, the Gram positive cell wall does not restrict the diffusion of hydrophobic ONPs [49].

**Figure 8:**
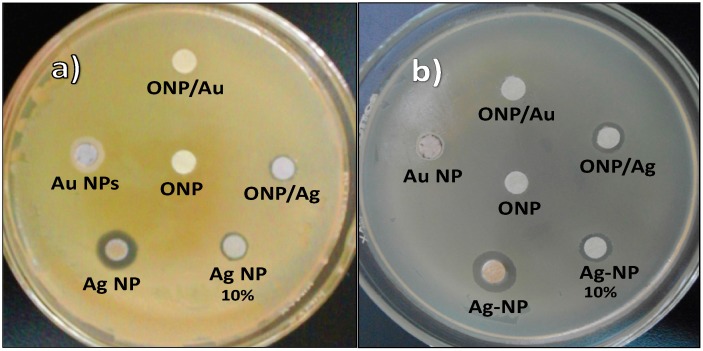
Disk diffusion test: (**a**) *Staphylococcus aureus*; (**b**) *Salmonella typhi.*

The disk diffusion data (Figure 8) show that there is no antibacterial activity with ONPs, probably because of the small size ONPs that can easily diffuse through agar plates, resulting in a low content of NPs in the medium.

Interestingly, bacterial growth decreased further when the strains were treated with ONPs/Ag (lower MIC values), showing a higher inhibitory effect against Gram negative strains. This suggests that the presence of Ag atoms on ONPs induces a strong antimicrobial action by facilitating the interaction between microbial cells and NPs. The MIC values determined for AgNPs (Table 1) are slightly smaller than those of ONPs/Ag; however, a very low amount of Ag was employed (ratio 10:1) to prepare ONPs/Ag, showing that there is a significant effect in bacterial inhibition.

AgNPs were tested at two different concentrations: 5.0 mM for all NPs, and 0.5 mM, representing the low amount of Ag in the ONPs/Ag (ratio 10:1). This lower concentration was considered in order to prove whether the bacterial effect was caused by Ag NPs or by a possible synergic effect of Ag and ONPs. The results (Figure 6 and Table 2) show that there is no significant difference in the diameter obtained at both concentrations, confirming that ONPs do not alter the antibacterial activity in solid medium.

**Table 2:** Antibacterial parameters for different bacteria treated with different NPs.

Inhibition Zone Diameter (mm) *					
Material	Concentration [44]	*Bacillus cereus*	*Staphylococcus aureus*	*Escherichia coli*	*Salmonella typhi*
ONPs	5.0	NP	NP	NP	NP
ONPs/Ag	5.0	7.0 ± 0.16	7.6 ± 0.12	9.3 ± 0.13	9.0 ± 0.10
ONPs/Ag	0.5	7.0 ± 0.11	8 ± 0.09	9.7 ± 0.12	9.3 ± 0.08
ONPs/Au	5.0	12.0 ± 0.21	11 ± 0.10	13 ± 0.14	13 ± 0.11
Ag-NPs	5.0	-	-	-	-
Au-NPs	5.0	-	-	-	-

* Inhibition zone diameter includes the paper disc diameter (6.0 mm) which contained stock solution (5.0 mM).

Although several possible mechanisms have been proposed to explain the toxicity of NPs to bacteria [50], we consider that the smaller size ONPs/Ag (<15 nm) first possibly adsorb onto the surface of the cell membrane, and then diffuse into the cell, hence affecting cell functions including replication and respiration [51]. Once inside the cell, Ag doped ONPs slowly oxidize to Ag^+^ ions and can inactivate proteins and/or intercalate between the purine and pyrimidine bases of DNA [52], and also bind with thiol groups of bacterial proteins, all of which lead to cell death [51,53,54,55]. Although the same antibacterial effect was expected for ONPs/Au as for ONPs/Ag, there is a decrease in the bacterial inhibition, even lower than that of ONPs. If the aggregation increases, the concentration of active NPs decreases, so the MIC value increases.

The reported toxicity of Ag^+^ and AgNPs [56] shows Ag^+^ ion to be more toxic than AgNPs, and the availability of soluble Ag^+^ is correlated with bacterial toxicity. The uptake of Ag^+^ ion is higher than of Ag because the direct uptake of AgNPs by cells is negligible. So, AgNPs are oxidized to Ag^+^, which then are taken up by cells. This was proved by using cysteine as ligand in the medium. The toxicity of Ag^+^ was analyzed in the presence and the absence of cysteine, which can coordinate with Ag^+^ to form a complex. In the presence of cysteine there is less soluble Ag^+^, resulting in a lower toxicity. In other reports, ROS can mediate between AgNPs and Ag^+^ via an electron-charging/discharging process [57,58] (superoxide reduces Ag^+^ to Ag [56], and H_2_O_2_ oxidizes AgNPs to Ag^+^). The ROS can also be produced under weak light (visible or IR). For example, the formation of ROS has been detected in the cell culture by EPR in visible light [59,60,61]; similarly, the generation of ROS has been found in the presence of infrared and visible light [62,63]. Although both AgNPs and Ag(I) are toxic to bacteria [64,65,66], and AgNPs cause cell death by damaging bacterial cell membranes by enhancing permeability and disturbing respiration [67], while Ag^+^ interacts strongly with the thiol groups of proteins [68] and severely disturbs bacterial membrane integrity thereby affecting DNA replication [69]. In addition, certain NPs can produce reactive oxygen species (ROS) through their reaction with oxygen [70,71] or light-involved generation of electron−hole pairs, causing the dissolution of NPs. However, there is no clarity in the mode of action with cells, and it requires further study.

In the present work, we analyzed the presence of soluble Ag^+^ in solution by adding NaCl solution to AgNPs suspension in order to verify whether Ag is present as NPs, or as Ag^+^, and only Ag present in AgNPs, was found. The bacterial growth inhibition (I, %) of different NPs was analyzed, and a synergistic effect for ONPs/Ag was found. The net inhibition (I) rate of Ag-ONPs is higher than the sum of ONPS and AgNPs as shown below:

I [Ag-ONPs] > (I [Ag] + I [ONPs])

The inhibition trend is as follows: ONPs/Ag > ONPs > ONPs/Au. ONPs/Ag exhibits a high inhibition to bacterial growth because of the synergistic effect, *i.e.*, the particles diffuse into cells through the membrane, then AgNPs is slowly oxidized to Ag^+^ causing severe damage to cell functions (Ag/Ag^+^, *E*_oxidation_ = −0.80 V); simultaneously, ONPs (LA) yields as redox reaction with the components, so both effects lead to a quick cell death. With ONPs/Au, the oxidation of stable Au to Au^+^ is more difficult (*E*_oxidation_ = −1.50 V) than for Ag due to its higher oxidation potential, so the Au^+^ ion present inside the cell is negligible; however, AuNPs bind strongly with ONPs through the disulfide group, inhibiting the interaction of the particles with cell components, leading to low bacterial inhibition growth. In the case of ONPs, it involves only redox reaction with the cell components, so its inhibition is less than ONPs/Ag but it is higher than ONPS/Au.

**Figure 9:**
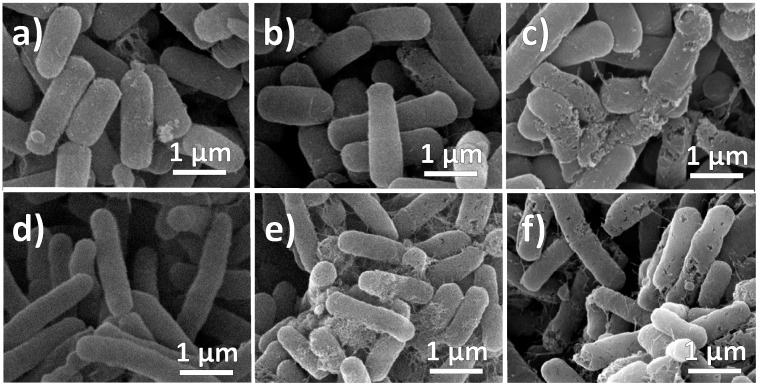
SEM images of bacterial strains after treatment with NPs. (**a**) *Bacillus cereus* with ONPs; (**b**) *Bacillus cereus* with ONPs/Ag; (**c**) *Bacillus cereus* with Ag NPs; (**d**) *Salmonella typhi* with ONPs; (**e**) *Salmonella typhi* with ONPs/Ag and (**f**) *Salmonella typhi* with AgNPs.

The antibacterial effect of the NPs on *E. coli* and *B. cereus* was also analyzed by SEM to determine possible morphological changes in the cell membrane (Figure 9). The results (Figure 9a,d) show that there is no visible damage to the cell membrane after treatment with ONPs and this observation was consistent with several repeated experiments. This suggests that the small sized ONPs (~5 nm) can easily diffuse through cell membranes without causing perceptible damage and still cause cell death.

However, many holes were produced in the membranes of cells treated with ONPs/Ag (Figure 9b,e), similar to the bacteria treated with AgNPs (Figure 9c,f). Generally, metal NPs bind onto the cell surface due to the electrostatic interactions between NPs and the cell membrane because bacterial surfaces are known to be negatively charged [72] and metal NPs or metal oxide NPs are known to have a slightly positive charge [73], causing the deformation of cell walls. This affects bacterial functions through the interaction with other bacterial components, leading to bacterial death.

### 2.3. Computational Procedure

The toxicity and redox properties of the LA is directly related to its geometrical and electronic properties; the variation of these parameters during its binding with Ag or Au is also important, thus full optimization was performed for the LA-structure by Density Functional Theory (DFT) using Gaussian’09 at B3LYP with 6-311+G(d,p) basis set [74]. Polarizable Continuum Mode (IEF-PCM) was used to see the solvent effect in THF/water as solvent. Structural data were used to calculate Time-Dependent (TD-DFT) parameters and molecular orbital energies (Table 3) for LA to determine the UV-Vis absorbance [75,76]. Hybrid functional B3LYP with LANLDZ basis set was employed to optimize the complex structure (LA-Ag), and then the UV-visible spectrum was obtained through TD-DFT calculations. The MO energy data are consistent with the experimental results that the participation of molecular orbitals in bonding such as HOMO, HOMO-1, HOMO-2 with Ag is greater than those with Au because a smaller energy difference between the orbitals (HOMO-HOMO-1, 0.272 eV; HOMO-1-HOMO-2, 0.517 eV) was observed for LA-Ag than for LA-Au (HOMO-HOMO-1, 0.822 eV; HOMO-1-HOMO-2, 0.601 eV). This is an agreement with Density of State (DOS) that the appearance of orbitals (energy gap between the orbital) more uniform in HOMO level for LA-Ag than for LA-Au, showing that the binding of LA with metal is important for the bacterial deformation. In addition, the molecular orbitals which are located within disulfide ring, facilitate strong interaction with metals, particularly with Ag (small energy gap of HOMO-LUMO, 2.24 eV) than Au atom (HOMO-LUMO, 2.43 eV). For the case of LA, which involves the bonding directly with the cell components, it is difficult to compare with the interaction of LA with metals than that of with the cell components. 

**Table 3:** Frontier molecular orbital (FMOs) for LA and its decorated with Ag or Au.

Molecular Orbital	Energy (eV)		
	LA	Ag-LA	Au-LA
LUMO+3	0.001	0.490	−0.288
LUMO+2	−0.027	0.005	−0.620
LUMO+1	−0.310	−0.599	−2.637
LUMO	−1.361	−1.551	−3.371
ΔE_(HOMO-LUMO)_	4.446	4.136	2.433
HOMO	−5.807	−5.687	−5.804
**ΔE_(HOMO-HOMO-1)_**	**1.951**	**0.272**	**0.822**
HOMO-1	−7.758	−5.959	−6.626
HOMO-2	−8.073	−6.476	−7.227
HOMO-3	−8.696	−7.456	−7.978

**Figure 10:**
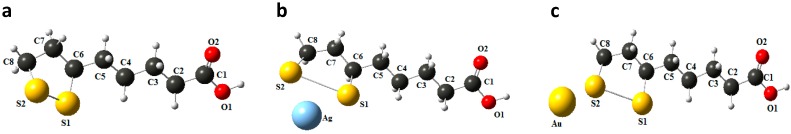
Optimized structures of LA (**a**) and its forms decorated with Ag (**b**) or Au (**c**).

The DFT study (Figure 10) shows that two sulfur atoms are coordinated with Ag to form the complex structure, which is consistent with the electrostatic potential analysis, where negative charge was detected for sulfur and carboxylic acid-oxygen of LA. This also agrees with the electron density analysis that high electron density S and O atoms can act as good receptors, and coordinate with a metal ion (acceptor).

**Figure 11:**
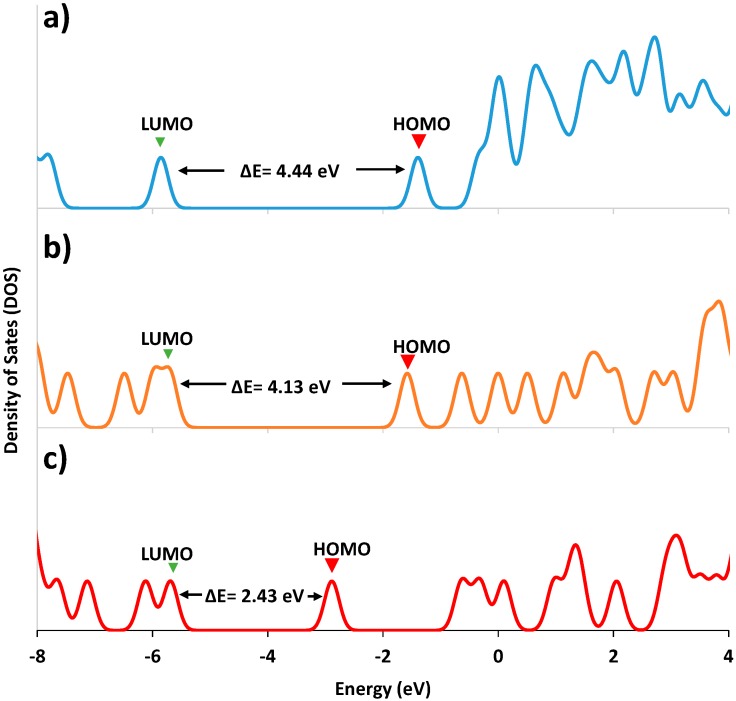
Density of states for LA, and its doping with metal atoms: (**a**) LA-ONPs; (**b**) Ag doped (1.0%) with LA-ONPs and (**c**) Au doped (1.0%) LA-ONPs calculated through B3LYP/6-311G(d,p) for LA and B3LYP/LANL2DZ for Ag/Au-LA.

**Figure 12:**
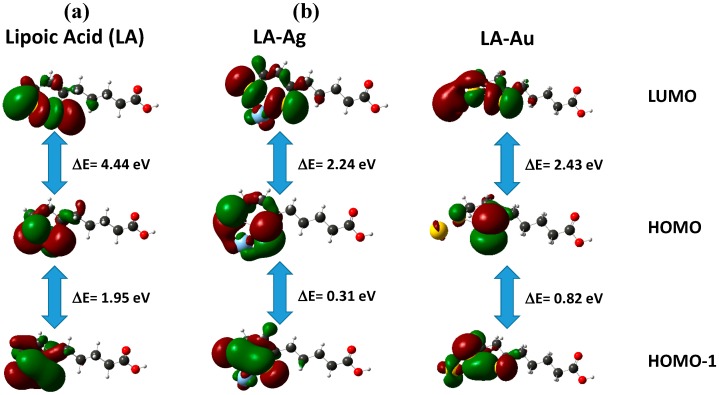
Frontier molecular orbitals: (**a**) LA; (**b**) LA/Ag.

Density of states (DOS) was determined by Energy-DFT for both LA and LA-Ag complex; the results (Figure 11) show that a close energy gap for orbitals was observed for LA-Ag and wider for LA. For LA, with a high density of states, the individual electronic states are fused to form a wide band above LUMO + 3 levels, while for LA-Ag, there are orbitals involved in bonding with the metal (Figure 12) [77,78], so only a few independent states appeared, yielding a small energy gap at high energy (E = 3.8 eV, LUMO + 15). 

## 3. Experimental Section

### 3.1. Materials and Methods

α-Lipoic acid, HAuCl_4_, AgNO_3_, and NaBH_4_ were used as received from Sigma-Aldrich (Mexico City, Mexico). Tetrahydrofuran (THF, J. T. Baker, Mexico City, Mexico) was employed to synthesize ONPs. The bacterial strains for cell culture were obtained from the Strain Bank (WFCC/WDCM-100) of the UNAM Faculty of Chemistry, and maintained in cysteine tryptic agar (CTA, BIOXON, Mexico City, Mexico). Mueller Hinton (DIBICO, Mexico City, Mexico) for inoculum dilution mixture, nutritive broth (DIBICO) for inoculum suspension and Petri dishes (90 × 15 mm) for cell culture and 96-wells plates were employed for the bacterial inhibition tests. Glutaraldehyde (25% purity) and absolute ethanol were used to prepare the samples for SEM analysis.

### 3.2. Equipment

All synthesized NPs were characterized by UV-Vis spectrophotometer (Perkin Elmer 25, Waltham, MA, USA, range 270 nm to 1100 nm), Transmission Electron Microscopy (TEM, JEOL 2010, Tokyo, Japan) with Energy Dispersive X-Ray Spectroscopy (EDS). For TEM and EDS analysis, samples were prepared by dropping the NPs solution on a 300 mesh copper grid, which was allowed to dry, and coated with a thin layer of carbon. The morphology of the biological samples was also examined with a Quanta 400F field emission scanning electron microscope (SEM, FEI, Hillsboro, OR, USA).

### 3.3. Experimental Procedures: Organic Nanoparticles (ONPs)

The LA ONPs of were prepared as reported previously [79]: α-lipoic acid (0.1 mM) dissolved in THF (1.5 mL) was slowly injected into deionized water (100 mL) under sonication to yield a turbid colloidal suspension of ONPs (10.0 mM).

### 3.4. Gold Doped-Organic Nanocomposites (ONPs/Au)

The colloidal solution of ONPs (50 mL, 10. 0 mM) was mixed with a solution of HAuCl_4_ (500 μL, 10 mM) to reach a final molar ratio of 10:1 (ONPs:Au). On addition of the Au^3+^ solution, the resulting solution turned red for a short time and then changed into a colorless solution. Finally, NaBH_4_ (200 μL, 10 mM) was added dropwise under vigorous stirring to yield the ONPs/Au. 

### 3.5. Silver-Organic Nanocomposites (ONPs/Ag)

ONP/Ag was prepared by the reduction method: the ONPs suspension (10.0 mM) was added to a AgNO_3_ solution (20.0 μL, 50.0 mM) in a molar ratio of 10:1 (ONPs:Ag), and then NaBH_4_ (150 μL, 10.0 mM) was added drop-wise under stirring. The turbid suspension turned to a pale yellow-orange color.

### 3.6. Gold and Silver Nanoparticles

Au NPs or Ag NPs were synthesized by using NaBH_4_ (10.0 mM) in deionized water, and the resultant colloidal solutions were red (AuNPs) or yellow (Ag NPs).

### 3.7. Standardization of Inoculum Suspension

Inoculum suspensions were prepared with 24 h-old cultures of bacterial strains on nutritive agar using sterile saline solution (0.9% *w*/*v*) and then the bacterial concentration was adjusted to 0.5 on the standard McFarland scale.

### 3.8. Antibacterial Studies

The antibacterial properties of the ONPs were analyzed against four bacterial strains, two of which were Gram positive (*Staphylococcus aureus* and *Bacillus cereus)* and the other two Gram negative (*Escherichia coli* and *Salmonella typhi*). The identity of the isolated bacterial strains was confirmed according to the microbiological guidelines [80]. The susceptibility of the bacterial strains with ONPs, ONPs/Ag, ONPs/Au, Ag NPs and Au NPs was evaluated qualitatively and quantitatively in order to compare the susceptibilities of simple and doped ONPs.

### 3.9. Microbiological Qualitative Assays

Antimicrobial activity of all NPs was examined in both solid and liquid culture media. The disk diffusion method [80,81] was employed in solid tests; Mueller Hinton agar plates were inoculated in the previously standardized inoculum suspension with using a sterile cotton-tipped swab; then, sterile paper discs (Whatman No.1, 6.0 mm in diameter) were impregnated with a 5.0 mM solution of the ONPs and were placed on the surface of the plates. The tests were carried out for ONPs/Ag, ONPs/Au, Ag NPs and Au NPs. Diameters of the growth inhibition zones were recorded in mm after incubating the plates for 24 h at 35 °C. Each experiment was carried out in triplicate.

Broth tests were performed by inoculating nutritive broth with bacterial strains (1 × 10^5^ UFC/mL) by adding a stock solution of test material (ONPs, ONPs/Ag, ONPs/Au, AgNPs and AuNPs) to achieve a final concentration of 10.0 mM in ONPs content. An inoculated tube without inhibitor was used as positive control. Bacterial cultures were incubated for 24 h at 35 °C, and then the optical density at 600 nm was measured. An aliquot of each diluted sample was inoculated onto a sterile Mueller Hinton agar plate in order to compare viable microorganisms after treatment with the NPs. 

### 3.10. Minimal Inhibitory Concentrations (MIC)

The modified microdilution method [82] was employed to determine the MIC for ONPs, ONPs/Ag, ONPs/Au, Ag NPs and Au NPs against different bacteria. Each well of microplates (96-wells) was filled with 0.1 mL of inoculated nutritive broth (final concentration 1 × 10^5^ UFC/mL) and 0.1 mL of nanomaterial solution at the respective concentration. Different solutions were prepared from 10.0 mM stock solutions of each material (ONPs, ONPs/Ag, ONPs/Au, Ag NPs and Au NPs) to give a range of concentration from 0.05 to 0.50 mM. Microplates were incubated overnight, and then 10.0 µL of 10.0 mM aqueous *p*-iodonitrotetrazolium chloride solution was added to all wells to measure bacterial growth as determined by the development of a red color by the biologically active cells. MIC can be defined as the lowest concentration of ONPs, ONPs/Ag, ONPs/Au, AgNPs and AuNPs that generates a colorless well, showing a completely inhibited bacterial growth.

### 3.11. Morphological Analysis of Bacterial Cells Treatment with NPs

The effect of NPs (ONPs, ONPs/Ag, ONPs/Au and AgNPs) on the morphology of bacterial cells was analyzed by Scanning Electron Microscopy (SEM) as described previously [83]. Bacterial samples, after exposure to MIC of each nanomaterial, were fixed with glutaraldehyde (2.5%) and then dehydrated with a series of ethanol solutions (30%, 50%, 70%, 90%, 95% and 100%). The dehydrated samples were dried by means of a critical point dryer (Auto- Samdri-815 Automatic Critical Point Dryer; Tousimis, Rockville, MD, USA). Each of the dried samples was placed onto SEM stubs and sputter-coated with gold using a cool-sputter coater (E5100 II, Polaron Instruments Inc., Hatfield, PA, USA). The cell damaged portions were then observed by using SEM at 20 kV secondary electrons, capturing the areas where morphological changes occurred in treated cells.

## 4. Conclusions

Three different organic and hybrid organic-metallic nanoparticles were tested as bacterial growth inhibition agents. The antibacterial studies showed that among all tested materials ONPs/Ag has the highest inhibition effect, while Au-containing materials revealed a low inhibitory effect. It was also shown that Gram positive bacteria are more susceptible to the inhibitory effect of the tested materials as compared to Gram negative strains. The order of bacterial cell growth inhibition was found to be ONPs/Ag > ONPs > ONPs/Au. The morphology of bacterial cells was analyzed before and after inhibition treatments showing that even though both ONPs and Ag-ONPs exert an effective growth control, only bacterial strains treated with silver nanoparticles showed membrane puncturing and perforation.

## Supplementary Materials

Supplementary materials can be accessed at: http://www.mdpi.com/1420-3049/20/04/6002/s1.
