# Supplementary material for: Organic-Inorganic Hybrid Nanoparticles for Bacterial Inhibition: Synthesis and Characterization of Doped and Undoped ONPs with Ag/Au NPs
Source: Molecules. 2015 Apr 7;20(4):6002–21. doi: 10.3390/molecules20046002 (PMC6272726; doi:10.3390/molecules20046002)
Supplement: Supplementary file 1 [file molecules-20-06002-s001.pdf]

Supplementary Materials

Material	Peak 1	Peak 2
	Average Size (nm)	
ONPs	1.80	54.96
Ag	29.27	138.30
Au	2.68	55.24
Ag-ONPs	100.90	---
Au-ONPs	204.19	---

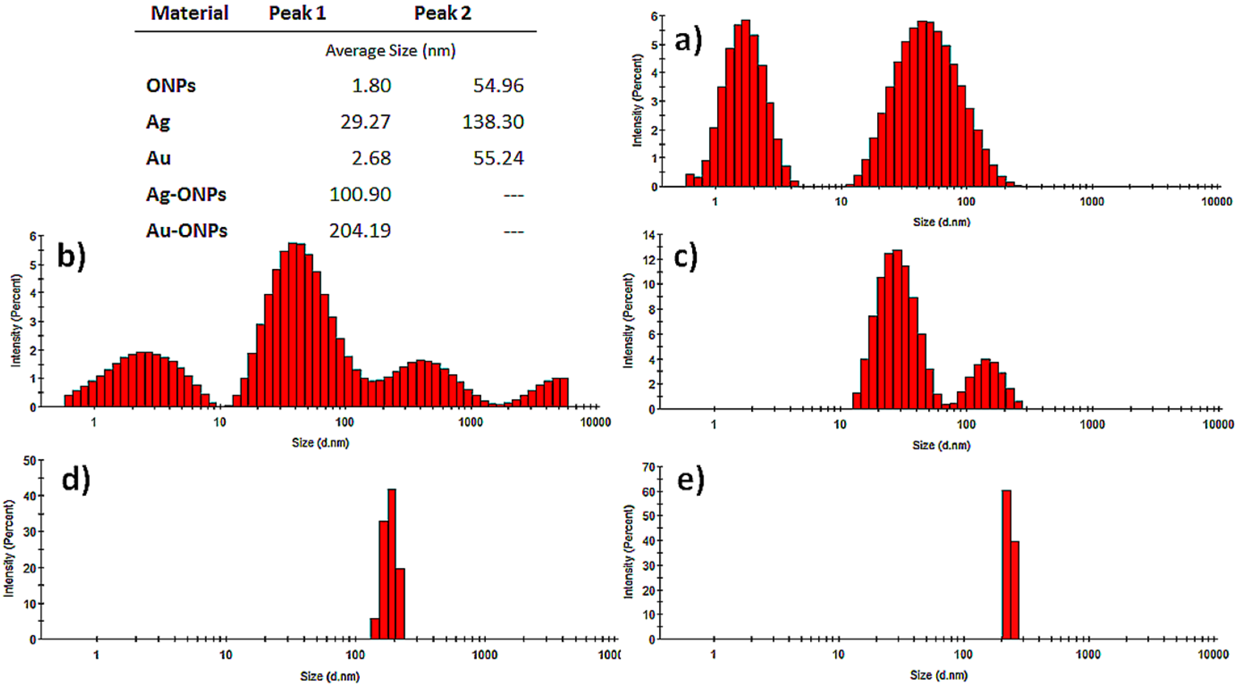

Figure S1. Dynamic Light Scattering (DLS) patterns for size distribution of different materials. (**a**) ONPs: (**b**) AgNPs; (**c**) AuNPs; (**d**) Ag-ONPs hybrid and (**e**) Au UNPs hybrid.

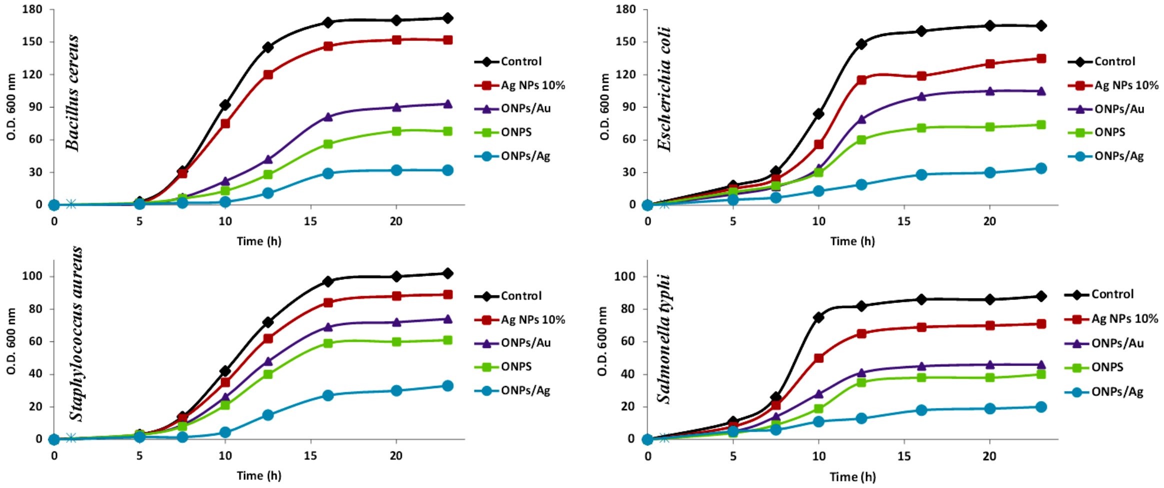

Figure S2. Effect of nanoparticles treatment over bacterial growth. Tested materials: ONPs (0.2 mM), ONPs/Ag (0.2 mM), ONPs/Au (0.2 mM) and Ag NPs (0.02 mM).

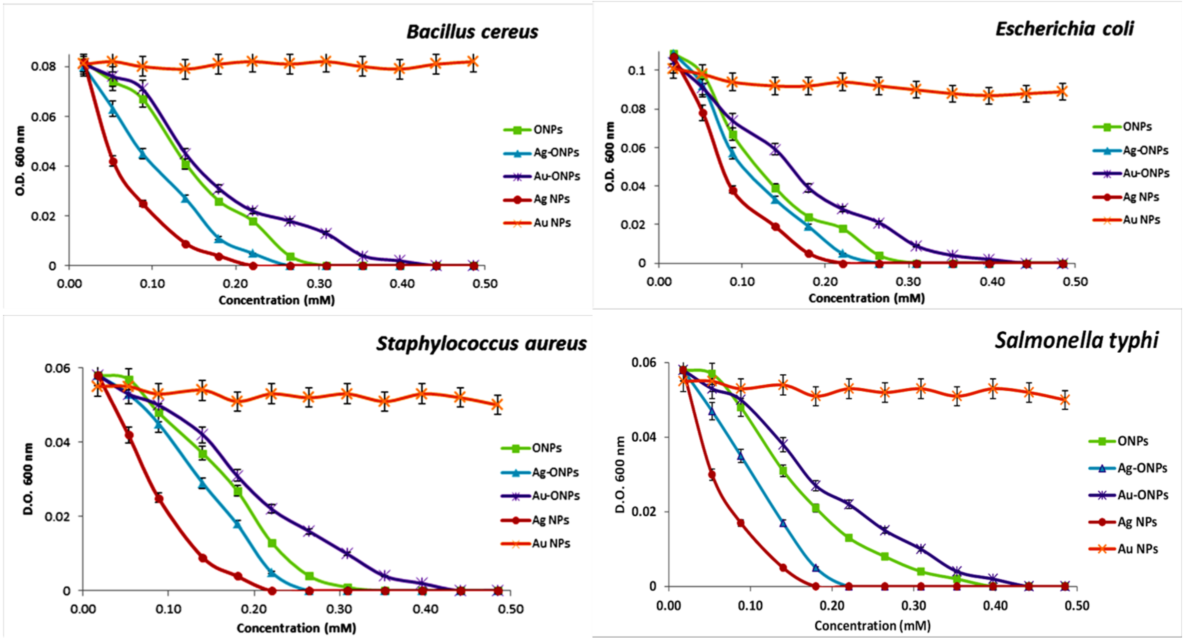

Figure S3. Effect of nanoparticles concentration on bacterial growth.

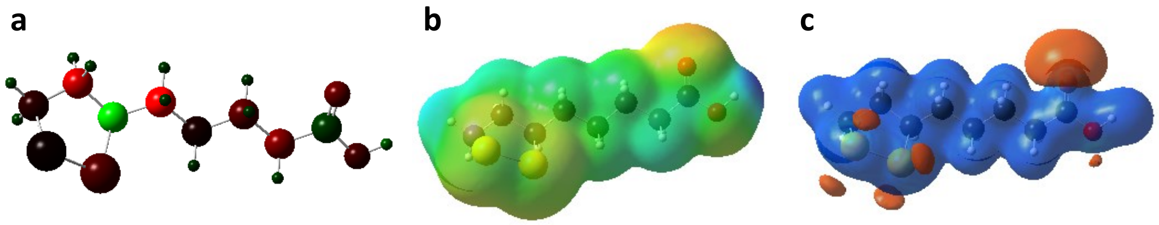

Figure S4. Theoretical (**a**) Mulliken Charges; (**b**) Electron density map; (**c**) Electrostatic potential map for Lipoic acid (LA) calculated though B3LYP/6-311G+(d,p).

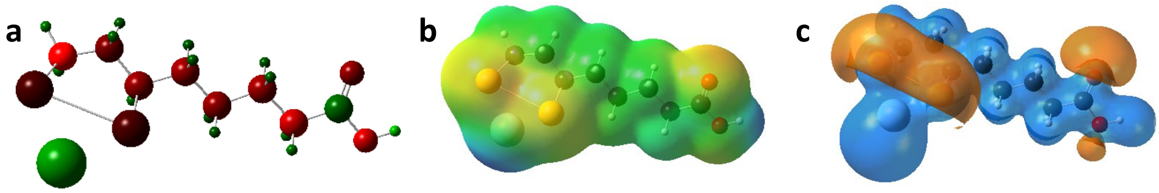

Figure S5. Theoretical (**a**) Mulliken Charges; (**b**) Electron density map; (**c**) Electrostatic potential map for Ag decorated Lipoic acid (LA-Ag) calculated though B3LYP/LANL2DZ.

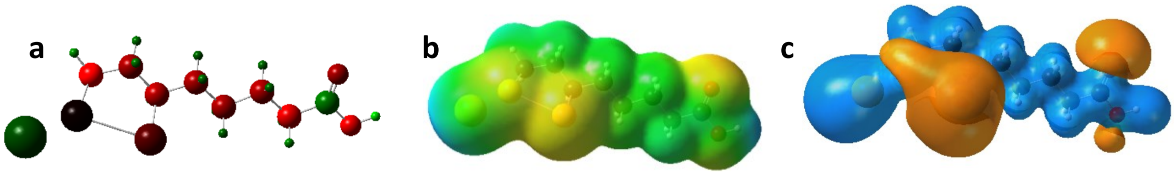

Figure S6. Theoretical (**a**) Mulliken Charges; (**b**) Electron density map; (**c**) Electrostatic potential map for Au decorated Lipoic acid (LA-Au) calculated though B3LYP/LANL2DZ.
